# Expression and Concentration of Matrix Metalloproteinase 9 and Tissue Inhibitor of Matrix Metalloproteinases 1 in Laryngeal Squamous Cell Carcinoma

**DOI:** 10.1155/2019/3136792

**Published:** 2019-04-18

**Authors:** Marlena Matulka, Aneta Konopka, Barbara Mroczko, Anna Pryczynicz, Andrzej Kemona, Magdalena Groblewska, Andrzej Sieskiewicz, Ewa Olszewska

**Affiliations:** ^1^Department of Otolaryngology, Medical University of Bialystok, Marii Skłodowskiej-Curie 24A, 15-276 Bialystok, Poland; ^2^Department of Neurodegeneration Diagnostics, Medical University of Bialystok, Waszyngtona 15A, 15-269 Bialystok, Poland; ^3^Department of General Pathomorphology, Medical University of Bialystok, Waszyngtona 13, 15-269 Bialystok, Poland; ^4^Department of Biochemical Diagnostics, Medical University of Bialystok, Waszyngtona 15A, 15-269 Bialystok, Poland

## Abstract

The aim of this study was to assess the expression of MMP-9 and TIMP-1 in cancerous tissue as well as in the serum and plasma concentrations of these proteins in patients with laryngeal cancer and compare the results to the inflammatory reaction in healthy subjects. Twenty-seven patients who were diagnosed with laryngeal carcinoma and selected for total laryngectomy were included in the study group. MMP-9 and TIMP-1 expression in tissues was assessed using immunohistochemical assays. Immunoenzymatic ELISA methods were used to measure MMP-9 and TIMP-1 concentrations in serum and plasma. MMP-9 and TIMP-1 were identified in tumor cells and in the tumor stroma compartment, as well as in macroscopically healthy mucous membrane. MMP-9 expression was more significant in tumor stroma than in the perimatrix of the mucous membrane (*p* = 0.047). TIMP-1 expression was significantly higher in the matrix and perimatrix of the mucous membrane than in cancer tissue (*p* = 0.0093) and the tumor stroma compartment (*p* < 0.0001). Expression of TIMP-1 was observed more frequently in tumors without infiltrated lymph nodes (*p* = 0.009). Serum concentrations of MMP-9 and TIMP-1 as well as plasma TIMP-1 concentration were significantly higher in the study group than in the control group (*p* = 0.0004, *p* = 0.002, and *p* = 0.0001, respectively). A significantly higher TIMP-1 level in plasma was found in patients with poorly differentiated tumors compared to G1 and G2 (*p* = 0.046). MMP-9/TIMP-1 rate in serum was significantly higher in the study group than in the control group. The balance between the level of MMP-9 and TIMP-1 is disrupted in laryngeal cancer. The significant correlation between TIMP-1 expression and the presence of lymph node metastases, as well as that between TIMP-1 plasma concentration and stage of cancer histological differentiation, might indicate the importance of this molecule as a prognostic factor during carcinogenesis.

## 1. Introduction

Laryngeal squamous cell carcinoma (LSCC) is one of the most common SCC of the head and neck. Despite improvements in diagnostic and therapeutic techniques, there has been no improvement in 5-year survival rates for laryngeal cancer patients over the last three decades [1].

Numerous studies have indicated that the extracellular matrix is the main “support” structure of the tissues, and it could be involved in the suppression of cellular migration and proliferation. The changes mediated by enzymes involved in extracellular matrix (ECM) degradation, such as metalloproteinases, may have an impact during carcinogenesis [2].

Matrix metalloproteinases (MMPs) are important proteolytic enzymes which participate in the degradation of components of the ECM and abundant macromolecules localized on the cell surface and take part in many physiological processes. There is evidence that there are changes in the MMP activity in many pathological conditions, such as inflammatory diseases and cancer [3]. Degradation of the ECM by MMPs is crucial for malignant tumor development and progression, because MMPs regulate cancer cell growth and proliferation, metastasis, angiogenesis, and the immune response to cancer [4]. The activity of MMPs is strictly regulated by their inhibitors [5].

Matrix metalloproteinase 9 (MMP-9) has an impact on cancer growth due to type IV collagen degradation, a major component of the basement membrane (BM). Loss of the continuity in the BM structure determines the beginning of the cancer cell migration from the primary tumor as it spreads from a local and regional disease to remote metastasis [5]. MMP-9 was shown to act as a controller of the tumor neovascularization [6].

Regulation of MMP activation is complex, and the process is only partially understood. The most important regulator of MMP-9 is a specific inhibitor of this molecule called tissue inhibitor of metalloproteinases 1 (TIMP-1) [2]. The primary known role of TIMP-1 is exerting an inhibitory effect on the catalytic activity of MMPs, due to the inactivation of the active forms of this family of enzymes. Recent studies have shown an association between the activity of TIMPs and tumor aggressiveness and poor prognosis. This effect might be due to the activation of matrix metalloproteinase 9 (MMP-9) and promotion of cell proliferation, inhibition of apoptosis, and regulation of angiogenesis [7].

The aim of this study was to assess the expression of MMP-9 and TIMP-1 in cancerous tissue as well as in serum and plasma concentrations of these proteins in patients with laryngeal cancer and compare the results to the inflammatory reaction in healthy subjects. Our hypothesis was that the expression and concentration of MMP-9 and TIMP-1 are associated with TNM and histological differentiation of cancer.

## 2. Materials and Methods

### 2.1. Patients

During the 2-year study, 44 patients were treated for laryngeal cancer. 27 out of 44 patients were included in the current study. The following are the inclusion criteria: ≥18 years of age, total laryngectomy, no prior radiotherapy and chemotherapy, and no T1 cases. All patients with any signs of either chronic or acute inflammatory diseases were excluded from the study. The study group included 27 patients (23 males and 4 females, aged 48-80 years, mean age 60.3) who underwent total laryngectomy. Histopathological examination of larynx tumor specimens revealed squamous cell carcinoma in all cases.

Stage of the disease, primary tumor, and lymph node involvement were determined as per the TNM classification. Study group patients were divided into two groups according to the lymph node involvement: all cases with lymph node involvement (N+) and cases without lymph node metastases (N-). Characteristics of patients in the study group are presented in Table 1.

The control group consisted of 25 patients (21 men and 4 women, aged 20-59, mean 42.0 years old), who were admitted to the hospital to undergo septoplasty due to nasal patency impairment and who had no other medical problems.

All patients signed a written consent form, which included an agreement to participate in the study and to allow taking samples of blood as well as the biopsy of tumor and adjacent tissue specimens during the surgery. The study was approved by the local Ethics Committee.

### 2.2. Material

Solid tissue specimens were obtained intraoperatively from 27 patients. During the primary surgical procedure, part of the resected tumor and part of the macroscopically healthy adjacent mucous membrane, minimum of 2-2.5 cm from the tumor, were included in the study. Tissue specimens were immediately fixed in 10% buffered formalin. During the following 48 hours, specimens were embedded in paraffin.

Prior to the surgery, venous blood samples were taken from patients with laryngeal carcinoma and from the control group. Blood samples were collected using the S-Monovette blood collection system. Venous blood samples with lithium heparin as an anticoagulant were instantly centrifuged to obtain plasma. To obtain serum, the blood samples were first left to clot before centrifugation. Serum and plasma samples were stored in Eppendorf tubes at -80°C until analysis.

### 2.3. Immunohistochemistry

Before immunohistochemistry was performed, each specimen, i.e., tumor and healthy mucous membrane, was stained with hematoxylin and eosin (H&E) to verify cancerous tissue and lack of tumor cells in specimens, respectively. An inflammatory reaction was estimated semiquantitatively as mild (+), moderate (++), and marked (+++) when the leukocytes occupied less than 10%, 10-50%, and 50% or more of the field of view (200x magnification), respectively. The regions with the highest density of inflammatory cells were assessed. Inflammatory cells localized in necrotic tissue were excluded from analysis.

Immunohistochemistry staining was performed on 4 *μ*m paraffin-embedded tissue sections. The sections were deparaffinized in xylene and hydrated in graded ethanol. To expose the studied antigens, the slides were heated for 20 min in EDTA buffer (pH = 9.0). Incubation of the sections in 3% hydrogen peroxide was performed to block endogenous peroxidase activity. The expressions of MMP-9 and TIMP-1 were examined using mouse monoclonal antibody matrix metalloproteinase 9 (clone 15W2, Novocastra, Leica Biosystems) and goat polyclonal tissue inhibitor of matrix metalloproteinases 1 (clone 6F6a, Novocastra, Leica Biosystems) as primary antibodies, respectively. These antibodies were applied at a dilution of 1 : 50 for 60 minutes at room temperature. The reaction was performed using a peroxidase detection system and localized with DAB as a chromogen (Novocastra, Leica Biosystems). Slides were then counterstained with hematoxylin, dehydrated, cleared, mounted, and then examined.

Two independent pathologists evaluating the immunohistochemical expression of the examined antigens were blinded to the patients' clinical data. They assessed MMP-9 and TIMP-1 expression in tumor and stroma cells, as well as in the matrix and perimatrix of healthy mucous membrane. The percentage of MMP-9- and TIMP-1-positive cells was calculated at a magnification of 400x in 500 cells in each sample. Staining results were evaluated using a semiquantitative score. Cells that showed cytoplasmic expression of MMP-9 or/and TIMP-1 were called positive cells. The expression was defined as follows: (1) negative (-), lack of immunoreactivity; (2) weak (+), positive immunohistochemical reaction in <10% of cells; (3) medium (++), 10-50% of positive cells; and (4) strong (+++), >50% of positive cells, according to Uloza et al. [8].

### 2.4. Enzyme Immunoassays of Serum and Plasma MMP-9 and TIMP-1

Serum and plasma MMP-9 and TIMP-1 concentrations were measured with enzyme-linked immunosorbent assay (ELISA) kits (Quantikine, R&D Systems, Abingdon, UK) according to the manufacturer's instructions. To determine MMP-9 concentration, serum samples were diluted 100-fold and plasma samples 40-fold. Serum and plasma samples were diluted 100-fold before analysis for TIMP-1.

### 2.5. Statistics

The statistical analysis was performed using GraphPad Prism 4.03 and STATA 11.0 statistical packages. Longitudinal data were checked for normality using the Shapiro-Wilk test and skewness test. The associations between MMP-9 and TIMP-1 expression and various clinical and histopathological features were evaluated by the chi-square test. Data of patients with laryngeal carcinoma were compared with those of the control group by the unpaired Student *t*-test for parametric data and the Mann–Whitney *U* test for nonparametric data. Three or more groups of data were compared using parametric or nonparametric ANOVA. Correlations were calculated using Spearman's test. The difference was accepted as statistically significant when *p* < 0.05.

## 3. Results and Discussion

### 3.1. Results

#### 3.1.1. Inflammatory Reaction Evaluation

**Figure 1:**
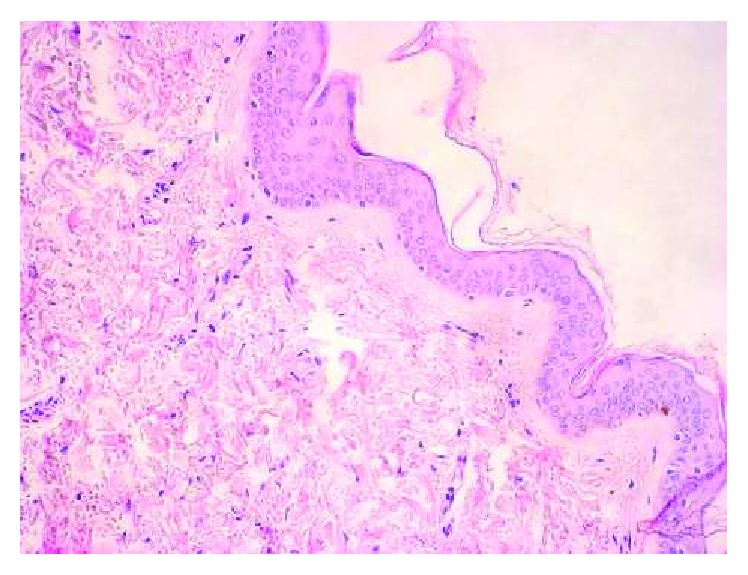
Healthy mucosa in the area of the tumor. H&E staining. Weak intensity (+) of the inflammatory reaction. Magnification ×200.

**Figure 2:**
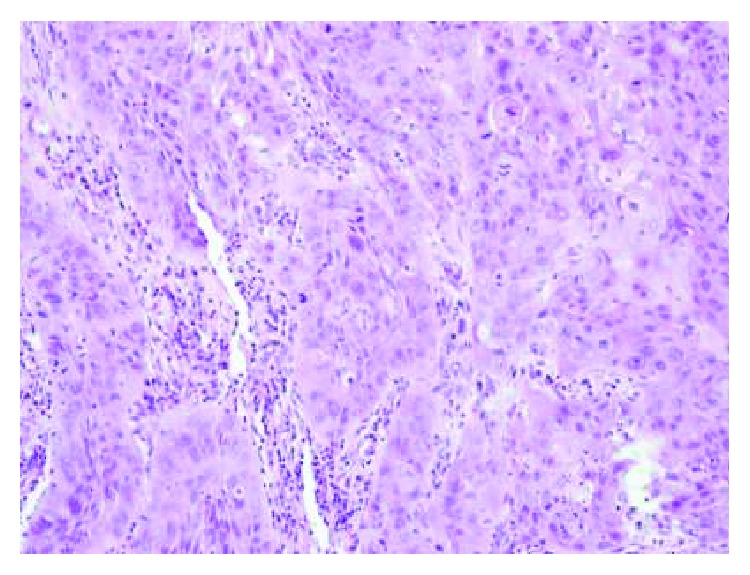
Squamous cell carcinoma of larynx cancer. H&E staining. Infiltration of inflammatory cells (i.e., lymphocytes, neutrophils, eosinophils, macrophages, and plasma cells) within the tumor stroma. Medium intensity (++) of the inflammatory reaction. Magnification ×200.

Inflammatory cell intrusion was statistically higher in laryngeal carcinoma than in macroscopically healthy mucous membrane surrounding the tumor (*p* = 0.002) (Figure 1). In laryngeal carcinoma tissue, inflammatory cells (i.e., lymphocytes, neutrophils, eosinophils, macrophages, and plasma cells) were mostly present in the stroma compartment (Figure 2). We did not observe a correlation between MMP-9 and TIMP-1 expression and intensity of inflammatory reaction.

### 3.2. Expression of MMP-9 and TIMP-1

#### 3.2.1. Laryngeal Squamous Cell Carcinoma Area

**Figure 3:**
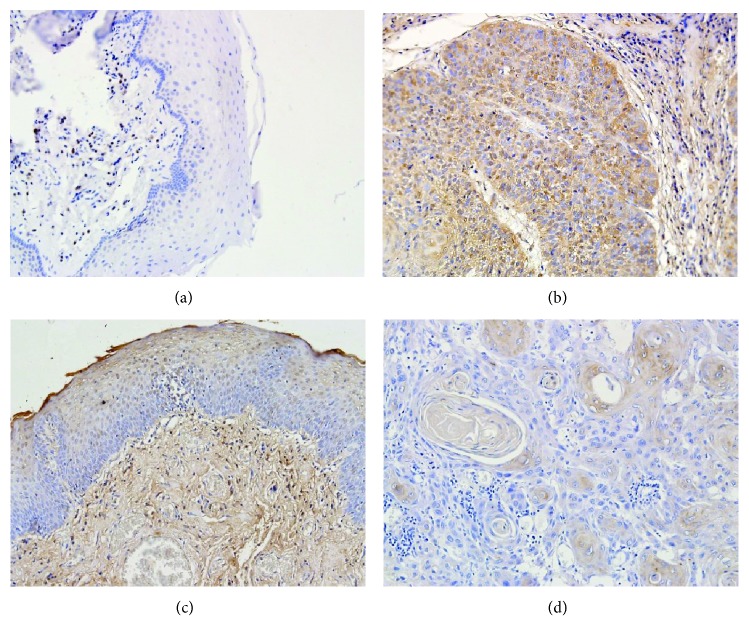
Immunohistochemical examination: (a) healthy mucosa of the larynx showing no reaction of MMP-9 protein in the matrix and weak expression in the perimatrix mainly in inflammatory cells; (b) strong cytoplasmic expression of MMP-9 in squamous cell carcinomas of the larynx and medium reaction in tumor stroma; (c) TIMP-1 expression in healthy tissue surrounding the tumor showing medium expression in the upper layer of the matrix and strong expression in the perimatrix; and (d) weak TIMP-1 expression in squamous carcinoma of the larynx. Magnification ×200.

In squamous cell carcinoma, the expression of MMP-9 observed in the stroma compartment was higher than that of the tumor cells. The MMP-9 expression was identified in the cytoplasm of the tumor cells in 34% of the examined specimens (only weak or medium expression) and in 96% of the tumor stroma compartment (mainly medium expression). The expression of TIMP-1 was observed in 46% of the examined specimens in tumor tissue and in 31% of the stroma compartment (Figure 3). MMP-9 expression was mostly present in the cytoplasm of expanding tumor stroma inflammatory cells, but there was no significant correlation between inflammatory reaction intensity and the degree of MMP-9 and TIMP-1 expression (*p* > 0.05).

#### 3.2.2. Control Group (Normal Mucosa)

MMP-9 and TIMP-1 expression was localized in the cytoplasm of matrix cells and in the perimatrix of the marginal compartment. Weak or medium MMP-9 expression was present in 81% of the matrix and in 100% of the perimatrix of normal mucosa. Statistical analysis revealed that MMP-9 expression was lower in the perimatrix of mucous membrane than in the tumor stroma (*p* = 0.047). Inversely, TIMP-1 expression was higher in the perimatrix of mucous membrane than in the tumor stroma (*p* > 0.0001). We also demonstrated a higher TIMP-1 expression in the matrix of normal mucosa in comparison to tumor tissue as well (*p* = 0.0093) (Table 2).

There was no significant difference in MMP-9 expression between tumor cells and normal epithelium (*p* > 0.05).

### 3.3. Expression of MMP-9 and TIMP-1 according to TNM Classification

There was no significant difference neither in MMP-9 nor in TIMP-1 expression in relation to the classification of the tumor (T), grade of the histological differentiation (G), or stage of laryngeal carcinoma. In 70% of advanced tumors (T3 and T4), there was no MMP-9 expression in the cancerous tissue, but 75% of the T4 tumors revealed medium or strong expression in tumor stroma. MMP-9 expression was present in all cases of the N(+) group (and it was assessed as medium or strong in 72% of the cases). In tumor stroma, we observed a significantly higher expression of TIMP-1 in the N(-) group in comparison to the N(+) group (*p* = 0.009).

### 3.4. Concentrations of MMP-9 and TIMP-1 in Serum and Plasma

**Figure 4:**
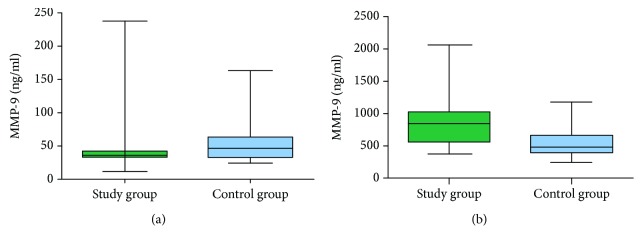
MMP-9 concentrations in serum (a) and plasma (b) of a study group and a control group.

**Figure 5:**
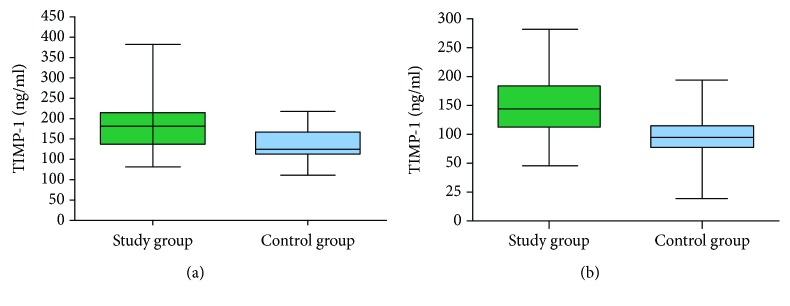
TIMP-1 concentrations in serum (a) and plasma (b) of a study group and a control group.

The concentration of MMP-9 in the study group was about 23-fold higher, whereas the concentration of TIMP-1 was about 2-fold higher in serum than in the heparinized plasma. There was a significant correlation between the serum and plasma concentrations of MMP-9 and TIMP-1 (*r* = 0.53, *p* = 0.004 and *r* = 0.58, *p* = 0.001, respectively). MMP-9 concentration in serum was significantly higher in the study group than in the control group (*p* = 0.0004). There was no statistically significant difference in the plasma enzyme concentration between the two groups (*p* = 0.109) (Figure 4). TIMP-1 concentration in serum as well as in plasma was higher in patients with laryngeal carcinoma than in the control group (*p* = 0.0020 and *p* = 0.0001, respectively) (Figure 5). No significant correlation was noted for both protein expression between the healthy tissue and the cancer specimen and their corresponding serum and heparinized plasma concentrations (*p* > 0.05).

### 3.5. MMP-9/TIMP-1 Ratio

**Figure 6:**
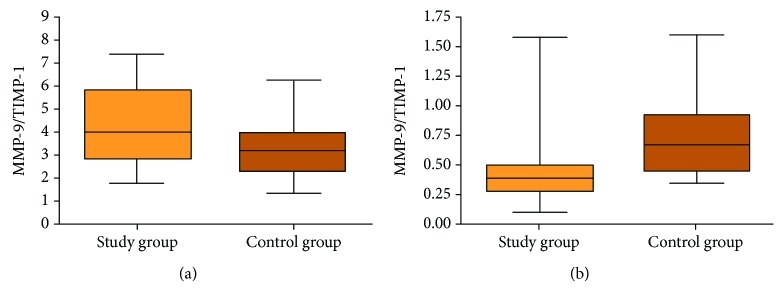
MMP-9/TIMP-1 rate in serum (a) and plasma (b) of a study group and a control group.

Serum MMP-9/TIMP-1 ratio was significantly higher in the study group in comparison to the control group (*p* = 0.0186) but lower in the heparinized plasma (*p* = 0.0010) (Figure 6).

### 3.6. Concentrations of MMP-9 and TIMP-1 according to TNM Classification

**Figure 7:**
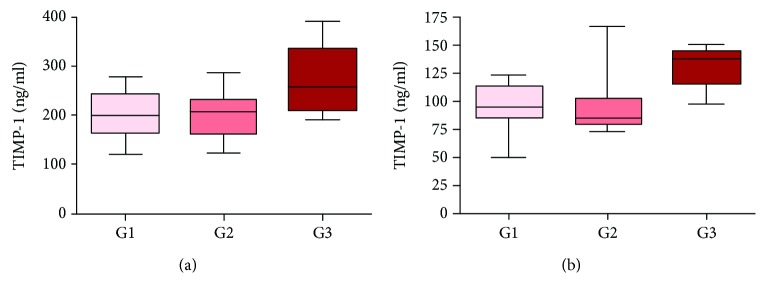
TIMP-1 concentrations in serum (a) and plasma (b) of G1, G2, and G3 tumors.

The tendency to have a higher MMP-9 serum concentration was observed in advanced clinical stages of cancer. We found a higher MMP-9 level in T4 tumors in comparison to those with the local spread of cancer (*p* > 0.05). A significantly higher TIMP-1 level in plasma was found in patients with poorly differentiated tumors compared to G1 and G2 (*p* = 0.046) (Figure 7). In higher levels of T cancer stages, a tendency for a higher TIMP-1 concentration was observed in both serum and plasma, but the difference was not statistically significant (*p* > 0.05).

## 4. Discussion

MMPs play an important role in normal biological as well as pathological processes, e.g., cardiovascular disease, arthritis, and otitis media with cholesteatoma [3]. Since the 80s, the significance of MMPs in the process of carcinogenesis was known. The expression of a group of metalloproteinases, including MMP-1, MMP-2, MMP-3, MMP-7, MMP-8, MMP-9, MMP-10, MMP-11, MMP-13, MMP-14, and MT-1 MMP [9], was shown in the head and neck squamous cell carcinomas. Gelatinases (MMP-2 and MMP-9) have been of special interest regarding carcinogenesis since those enzymes are involved in the degradation of collagen type IV in BM. Some researches indicate that these molecules may be candidates for tumor targeting genes in the gene therapy of human LSCC. The findings of Sun et al. [10] in mice demonstrate for the first time that the suppression of MMP-9 expression by lentivirus-mediated RNA interference can significantly inhibit invasion, growth, and proliferation of LSCC.

Data concerning the expression of MMPs and TIMPs in laryngeal cancer as well as the concentrations of MMPs and TIMPs in the serum and plasma obtained from laryngeal cancer have not been described yet. Therefore, the goal for our study was to evaluate the MMP-9 expression in laryngeal cancer and adjacent normal tissues with the correlation to blood concentrations in order to achieve a better knowledge of the role of MMP-9 and its inhibitor in laryngeal carcinoma.

There was high variability in the expression of MMP-9 in previous publications. Colović et al. [11], who studied 196 patients with laryngeal carcinoma, noticed MMP-9 expression in 48.4% cases. Wittekindt et al. [12] have found MMP-9 expression in 67.5% of studied specimens and Liu et al. [13] in 73.6% of all cases. Our study revealed MMP-9 expression in 34% of laryngeal carcinoma tissues in tumor cells and in 96% of cases in stromal compartment cells.

It was previously noticed that MMPs were synthesized by the tumor cells to degrade the surrounding extracellular matrix. Cells from the tumor stroma compartment produce most of the MMPs during carcinogenesis as well. Moreover, some researches indicate that it is not the malignant cells, but rather the stroma cells activated by tumor cells, which are the main source of that protease [9]. In Uloza et al.'s study, immunostaining for MMP-9 was detected in all studied glottic SCC cases, both in tumor cells and stroma. The expression of stroma MMP-9 was statistically significantly higher in the glottic SCC patients' group than in the benign vocal fold lesion group, which includes vocal fold polyps, recurrent respiratory papillomatosis, and laryngeal keratosis [14]. It is considered that MMP overexpression in tumor stroma is a host response to the presence and activity of transformed cells [2]. Increased proteolytic enzyme expression in the stroma of the cancer cells might in consequence limit the ability to synthetize MMPs by the neoplastic cells and lead to the loss of their differentiation [15]. The results of our study are consistent with the present theory on the cellular origin of MMP-9, which claims that the tumor stroma is the main source of this protease. In the present study, we recognized medium and strong expressions of MMP-9 in most tumor stroma cells and in the inflammatory cells infiltrating this compartment. To determine whether inflammatory cell infiltration has significant influence on the expression of MMP-9, we also studied the degree of inflammation in the examined tissue. We found that the level of inflammatory cell infiltration was significantly higher in laryngeal cancer tissue than in healthy tissue specimens. However, we have not found any significant correlation between the degree of inflammation and the expression of MMP-9 and TIMP-1.

There are studies that have not shown the MMP-9 expression in healthy tissue surrounding tumor [12]. Other data indicated significantly higher protein expression in the laryngeal carcinoma tissue but lower expression in normal mucosa [16]. Bodnar et al. [2] found MMP-9 and TIMP-1 in the cytoplasm of normal epithelium and in the stroma in the marginal compartment. They have shown statistically higher MMP-9 expression in the marginal stroma in comparison to the tumor stroma. In our study, we have also found the expression of MMP-9 and TIMP-1 in macroscopically healthy mucous membrane. The positive expression of the studied antigens was shown in the matrix as well as in the perimatrix. MMP-9 and TIMP-1 expression in the normal mucous adjacent to the tumor might be an effect of the activity of neoplastic cells and their interaction with the adjacent environment as well as inflammatory cell intrusion, which was shown in more than half of the studied specimens. In our study, the comparison of MMP-9 expression in the tumor stroma and in the perimatrix of the mucous membrane has demonstrated a statistically higher expression of the studied protein in the tumor stroma than in the perimatrix of healthy mucosa. TIMP-1 expression was higher in the matrix and perimatrix of normal tissue than in carcinoma tissue and tumor stroma, respectively.

Similar to MMP-9, in most studies, a positive expression of TIMP-1 was noticed. It was shown in neoplastic cells as well as in stromal cells [17]. Studies report different TIMP-1 expression levels in laryngeal carcinoma. Krecicki et al. [18] reported TIMP-1 expression in neoplastic cells and in tumor stroma in 72% of all studied cases, but they did not find this protein expression in normal tissue. Pietruszewska et al. [19] reported TIMP-1 expression in 25.3% of the studied cases. Cao et al. [16] and Christopoulos et al. [20] revealed TIMP-1 expression in laryngeal carcinoma tissue as well as in macroscopically healthy mucosa, but the expression level in malignant tumor was significantly higher than in normal tissue. In our study, positive TIMP-1 expression was observed more frequently in neoplastic cells than in tumor stroma cells. Surprisingly, we have found TIMP-1 expression in all studied cases of healthy mucous membrane, and the expression level in the matrix and perimatrix of mucosa was significantly higher than that in cancerous tissue and tumor stroma, respectively. It might, at least partially, be explained by the theory offered by Christopoulos et al. [20] that this observation might be due to the overall final activity of gelatinases that should be higher in the macroscopically normal specimens than in cancerous tissue to prepare the tissue for cancer invasion.

Most researchers have consistent results showing the enhanced expression of MMP-9 in laryngeal carcinoma, but they are not consistent about the relationship between MMP-9 expression and clinical and histopathological features or outcomes of the studied cancers [21].

A Croatian study on a large group of patients (*n* = 196) revealed high MMP-9 expression in patients with advanced clinical stage, in patients with metastases to the regional lymph nodes, and in patients with relapsed carcinoma [11].

Similar to the results obtained by others, we have not found a correlation between MMP-9 expression in cancerous tissue in tumor stroma and the T stage of the TNM classification. Some researchers [22–24] have shown a positive correlation between MMP-9 expression and metastases in regional lymph nodes. Other studies [12, 13, 25, 26] similar to ours did not confirm such correlation.

Colović et al. [11], Wittekindt et al. [12], and Akdeniz et al. [26] found higher MMP-9 expression in poorly differentiated tumors. According to Wittekindt et al. [12], this is evident especially on the early stage of tumor progression, when the neoplastic cell possesses a higher degree of differentiation. Our study did not show significant differences between MMP-9 expression and the grade of histological differentiation, which is consistent with other studies [11, 13, 16, 22, 25].

Gou et al. [22] and Papadas et al. [27] showed a higher MMP-9 expression in the advanced clinical stage, whereas Christopoulos et al. [20] revealed that protein expression in the cytoplasm of tumor cells decreased with the elevation of the clinical stage of laryngeal carcinoma. In our study, we have not found significant differences in MMP-9 expression in cancerous cells and tumor stroma as per the clinical stage.

In the case of TIMP-1, some researchers have revealed a significant correlation between protein expression and the grade of histological differentiation; however, such results are not consistent with other reports. For example Pietruszewska et al. [19] have shown a higher TIMP-1 expression in patients with poorly differentiated tumors, whereas Krecicki et al. [18] have observed this protein expression more frequently in G1 and G2 tumors. In our study, we have not found a significant differentiation in that feature.

Krecicki et al. [18] and Bodnar et al. [2] showed that TIMP-1 expression was higher in cases without lymph node metastases, whereas the lack of or a weak protein expression was connected with the enhanced risk of lymph node metastases. This data is consistent with the outcomes of our study, where a significant difference between TIMP-1 expression and lymph node status was also demonstrated.

Similarly to other authors, we found no differences in TIMP-1 expression and local tumor (T stage) or clinical stage of cancer [1, 16].

Most studies on the role of MMP-9 and TIMP-1 are related to the evaluation of the expression of these proteins in larynx cancer compared to the controls. There are only a few studies available where the MMP-9 level in blood was examined in the laryngeal carcinoma patients.

Hong et al. [28] who have examined the MMP-9 concentration in serum obtained from 50 patients diagnosed with head and neck SCC and Ranuncolo et al. [29] who have examined enzyme activity in plasma obtained from 91 patients with that group of carcinomas have shown a significantly higher MMP-9 level in the studied group compared to controls. Those authors have not found an association between the enzyme level and the clinical and histological features of the tumors [28].

In the relevant literature, we have not found any studies where the MMP-9 concentration in serum or plasma obtained in an isolated laryngeal carcinoma group was measured using immunoenzymatic assays. Klisho et al. [24] have examined MMP-9 and TIMP-1 activity in serum obtained from patients with that localization of tumor and revealed statistically significant differences in TIMP-1 but not in MMP-9 activity between the study and the control groups. They did not find a significant correlation between MMP-9 and TIMP-1 activity, consistent with our study results. However, in our study a significantly higher concentration of MMP-9 in serum was observed in a study group compared to a control group. We have not noted a significant relation between MMP-9 concentration and clinical or histological features.

It is considered that MMP-9 concentration in serum is significantly higher compared to the enzyme concentration examined in heparinized plasma. In our study, MMP-9 concentration was 23-fold higher than the level obtained in plasma in a study group. It is considered that such a result is due to some additional portion of MMP-9 released from activated platelets and leukocytes in serum [30]. This is the reason why heparinized plasma, rather than serum, is preferable material to examine MMP-9 concentration using the immunoenzymatic assay.

In our study, TIMP-1 concentration in serum as well as in plasma was significantly higher in the study group compared to the control group. The analysis of TIMP-1 concentration in plasma revealed a significantly higher level in the case of poorly differentiated tumors than in G1 and G2 cancers. Choosing between serum and plasma for the best TIMP-1 and MMP-9 concentration assessment is still problematic and remains to be explained.

A decrease or lack of increase in TIMP expression during carcinogenesis confirms the theory that the formation of neoplasm influences the equilibrium between MMPs and TIMPs, with the domination of proteolytic enzyme activity. In the situation when MMPs enhance tumor growth and invasion, it is expected that TIMP expression will be inversely correlated with these processes. However, the results of studies in this field are often inconsistent and sometimes contradictory [19]. Krecicki et al. [18] revealed that the loss of equilibrium between MMPs and TIMPs, and especially inhibition disturbance, might be significant for the formation of lymph node metastases in laryngeal cancer patients.

In our study, we assessed the MMP-9/TIMP-1 ratio determined from the protein concentration in serum as well as in plasma. The significantly higher value of the ratio in the serum of patients with laryngeal carcinoma compared to the control group was obtained. In plasma, its value, inversely to serum rate, was lower in patients with laryngeal carcinoma than in the control group. The high serum index in patients with laryngeal cancer is a result of the high MMP-9 concentration in that group. The lower plasma ratio in cancer patients is determined by the high TIMP-1 concentration and relatively low MMP-9 concentration in comparison to the control group.

## 5. Conclusions

This study demonstrated for the first time the tissue expression and the concentrations of MMP-9 and TIMP-1 in serum and plasma in patients with laryngeal carcinoma. In laryngeal carcinoma, MMP-9 is mainly produced by stroma and tissues surrounding the tumor, which proves the interactions among cells and prepares the surrounding tissues for the tumor invasion. The significant correlation between TIMP-1 expression and the presence of lymph node metastases, as well as that between TIMP-1 plasma concentration and stage of cancer histological differentiation, might indicate the importance of this molecule as a prognostic factor during carcinogenesis. The balance between the level of MMP-9 and TIMP-1 is disrupted in laryngeal carcinoma. Further investigations to study the potential diagnostic and prognostic role of MMP-9 and TIMP-1 are needed, including larger cohort studies working on the hypothesis that these molecules could potentially help to identify high-risk patients with laryngeal carcinoma who require a more aggressive approach in therapy.

## Figures and Tables

**Table 1:** Characteristics of patients with laryngeal cancer.

Characteristic	No. of cases	%
Age	60.3 ± 9.11	—
Gender		
Male	23	85.2
Female	4	14.8
T category		
T1	0	0
T2	6	22.2
T3	12	44.4
T4	9	33.3
N category		
(-)	20	74.1
(+)	7	25.9
Clinical stage		
I	0	0
II	6	22.2
III	10	37.0
IV	11	40.7
Tumor differentiation		
G1	12	44.4
G2	11	40.7
G3	4	14.8

**Table 2:** MMP-9 and TIMP-1 expression in larynx cancer and adjacent mucous membrane (no. (%)).

Localization	MMP-9	TIMP-1	MMP-9	TIMP-1	MMP-9	TIMP-1	MMP-9	TIMP-1
	(-)		(+)		(++)		(+++)	
Tumor	17 (66)	14 (54)	5 (19)	3 (12)	4 (15)	5 (19)	0 (0)	4 (15)
Stroma	1 (4)	18 (69)	9 (35)	4 (15)	10 (38)	2 (8)	6 (23)	2 (8)
Matrix	4 (19)	0 (0)	16 (76)	5 (28)	1 (5)	12 (67)	0 (0)	1 (5)
Perimatrix	0 (0)	0 (0)	13 (62)	0 (0)	8 (21)	11 (58)	0 (0)	8 (42)

## Data Availability

The datasets used and analyzed during the current study are available from the corresponding author on reasonable request.

## Abbreviations

BM: Basement membrane
ECM: Extracellular matrix
ELISA: Enzyme-linked immunosorbent assay
H&E: Hematoxylin and eosin
LSCC: Laryngeal squamous cell carcinoma
MMPs: Matrix metalloproteinases
SCC: Squamous cell carcinoma
TIMP: Tissue inhibitor of metalloproteinases.
